# A retrospective multi-institutional assessment of breast radiology and pathology report readability and a novel patient tool (MedEd) utilizing American literacy standards

**DOI:** 10.1007/s11764-025-01842-0

**Published:** 2025-06-30

**Authors:** Samantha Greenseid, Gande Li, Olivia Mihulka, Arianna Vazquez, Alex Verosky, Salvador Rodriguez Franco, Samhita Bheemireddy, Madeline G. Higgins, Sarah Leslie, Sarah Tevis, Kristin Rojas

**Affiliations:** 1 Department of Surgery, Division of Surgical Oncology, University of Colorado Anschutz Medical Campus, Aurora, CO USA; 2 Department of Surgery, Division of Surgical Oncology, University of Miami Miller School of Medicine, Miami, FL USA; 3 Sylvester Comprehensive Cancer Center, University of Miami Miller School of Medicine, Miami, FL USA; 4 General Surgery Residency, Sky Ridge Medical Center, Lone Tree, CO USA; 5 University of Miami Miller School of Medicine, Miami, FL USA; 6 Albany Medical Center, Albany Medical College, Albany, NY USA

**Keywords:** Readability, Breast cancer, Pathology, Radiology reports, MedEd patient education tool

## Abstract

**Background:**

Health literacy is a critical factor in patient engagement and outcomes during cancer survivorship. With the 21st Century Cures Act, patients now receive immediate access to radiology and pathology reports, yet these documents often exceed the 6th-grade reading level recommended by the American Medical Association. While prior studies have examined general breast cancer materials, the readability of individualized clinical reports remains unexplored.

**Methods:**

In this multi-institutional retrospective study, 120 de-identified breast radiology and pathology reports from the University of Colorado and the University of Miami were analyzed across six report types. Readability was assessed using six validated indices. Additionally, 98 MedEd definitions and 96 MedEd-directed online patient education materials (OPEMs) were evaluated. MedEd is a Chrome plug-in designed to highlight key breast health terms and provide physician-curated definitions with links to vetted educational content.

**Results:**

Across institutions, most clinical reports exceeded a high school reading level, with surgical pathology reports being the least accessible. MedEd definitions and OPEMs demonstrated better readability but still surpassed the recommended 6th-grade level.

**Conclusions:**

Breast cancer–related reports and educational materials remain difficult for many survivors to comprehend. Improved design of patient-facing tools—potentially through AI integration and patient co-development—may enhance accessibility, empowerment, and shared decision-making.

**Implications for Cancer Survivors:**

This study underscores a critical gap between the readability of breast radiology and pathology reports and the literacy levels of many cancer survivors. As patients gain immediate access to these documents through electronic portals, the complexity of medical language can lead to confusion, anxiety, and disengagement from care. Survivors may be particularly vulnerable during post-treatment surveillance when understanding follow-up imaging and pathology is vital for shared decision-making. Tools like MedEd—while promising—must continue to evolve to meet national literacy standards. Simplifying medical reports and integrating patient-centered, Al-supported education tools can enhance survivors’ comprehension, foster autonomy, and ultimately improve their confidence and outcomes throughout survivorship.

## Introduction

Health literacy plays a crucial role in patient health, with poor literacy directly impacting health outcomes [1]. This is particularly relevant in the context of individual radiology and pathology reports. Since the passage of the 21 st Century Cures Act in 2016, which grants patients immediate access to their personal health information, there has been a significant increase in patient awareness and interest in their medical records [2]. However, a critical issue remains—patients often struggle to understand the complex medical terminology in their reports. A study by Mityul et al. found that patients and radiologists have differing perceptions of common language used in reports, contributing to confusion and widening health literacy gaps. [3]

To address this challenge, the American Medical Association (AMA) recommends that written patient materials be at or below a 6 th-grade reading level to align with general public health literacy [4]. However, research has consistently demonstrated that breast cancer–related materials exceed this standard [4]. The elevated readability of patient reports highlights a critical risk in supporting patient comprehension of their personal health information and patients often turn to the internet to understand their reports [5].

Online patient education materials (OPEMs) have become an increasingly popular resource for women seeking breast cancer information [6]. A national survey found that 72% of Internet users searched for health information online in the past year, with approximately 80% of these searches starting with a search engine such as Google, Bing, or Yahoo [7]. However, while OPEMs can serve as valuable tools, their high readability level may hinder patient understanding. A 2024 systematic review and meta-analysis evaluating breast cancer readability studies reported that breast online patient education materials (OPEMs) have an average readability level equivalent to the 12 th grade, which poses a significant barrier for patients with lower literacy levels [5].

The matter of readability is particularly pressing in the context of individual breast radiology and pathology reports, which patients often receive through patient portals hours to days before speaking with their provider. Without immediate provider guidance, patients may turn to unreliable sources or struggle with misinterpretation due to poor health literacy [8]. Despite the importance of this issue, there are no published studies assessing the readability of individualized breast pathology and radiology reports. Given the growing emphasis on personalized breast cancer care, improving the accessibility of these reports is essential for enhancing patient knowledge, facilitating shared decision-making, and promoting effective communication between patients and providers.

Previous research has explored patient understanding of breast pathology reports using survey-based methods. Verosky et al. found that most patients struggled with medical terminology, with only 1% correctly defining terms like “high grade” [12]. Another study assessed patients’ comprehension of their mammogram reports before and after conducting an internet search, revealing wide variability in interpretation and prompting recommendations for integrating patient educational materials into reports [13]. A broader study analyzed the readability of over 100,000 radiology reports from a large US health system using similar methods to ours, finding that only 4.2% of reports were written at or below an 8 th-grade reading level [14]. The current study builds upon this work by evaluating readability against the AMA’s recommended 6 th-grade level and focusing specifically on breast radiology and pathology reports.

We aimed to assess the readability of various de-identified breast radiology and pathology reports from the University of Colorado and the University of Miami. Additionally, we evaluated the readability of definitions and reliable breast OPEMs associated with MedEd terms, a novel patient education engagement tool designed for download on patient’s personal computers.

## Methods

### Study design

This study employed a retrospective, multi-institutional content analysis methodology to evaluate the readability of breast radiology and pathology reports, medical definitions generated by the MedEd browser extension, and associated online educational materials. Rather than a traditional observational study, this project utilized a document analysis framework focused on evaluating clinical documentation and digital health communication tools. The methodology aligns with previous health communication and readability assessment studies and adheres to guidance for structured reporting of instrument-based research.

### Data collection

A total of 120 de-identified breast imaging and pathology reports were collected from adult patients (≥ 18 years old) seen between January 2023 and the study period at two academic institutions: the University of Colorado Anschutz Medical Campus and the University of Miami Miller School of Medicine. Each institution contributed 60 reports, with 10 from each of six report categories: screening mammograms, diagnostic mammograms, breast ultrasounds, breast MRIs, percutaneous biopsies, and surgical pathology reports. Reports were retrieved via retrospective chart review and stored on encrypted servers in compliance with institutional data security policies.

MedEd, a Chrome browser extension developed at the University of Colorado, was used to generate layperson-friendly definitions for complex medical terms present in the reports. Additionally, 96 online patient education materials (OPEMs) hyperlinked through MedEd were analyzed to assess the readability of external patient-directed resources.

### Readability indices

Readability was assessed using the Readable.io platform, which integrates six validated and widely used indices:

1. Flesch-Kincaid Grade Level estimates US school grade level based on sentence length and syllables per word [23].
2. Flesch Reading Ease generates a score from 0 to 100 based on sentence and word complexity, with higher scores indicating easier readability [24].
3. Gunning Fog Index estimates grade level using sentence length and percentage of complex words [25].
4. Coleman-Liau Index uses characters per word and sentence length to determine readability [26].
5. SMOG Index, developed for health contexts, estimates grade level based on polysyllabic word count in a 30-sentence sample [27].
6. Automated Readability Index (ARI) calculates readability using character and word counts [28].

Each index estimates the US grade level or ease of comprehension based on text characteristics such as sentence length and word complexity. Scores were categorized as follows: ≤ 6 th grade (elementary), 6.1–8.9 (middle school), 9.0–12.0 (high school), and ≥ 12.1 (college or above).

### Data analysis

Descriptive statistics, including medians, interquartile ranges, and frequency distributions, were calculated for all readability scores. Comparisons were made between institutions and report types. Chi-square tests were used to compare observed distributions against the recommended AMA readability standard (≤ 6 th grade). Statistical significance was defined as *p* < 0.05. All analyses were reviewed by an institutional biostatistician to ensure methodological accuracy and reproducibility.

### Ethics approval

The study received ethical approval from both the University of Colorado IRB (#23–00123) and the University of Miami IRB (#2023–0765). All data were de-identified, and no patient interaction occurred.

### Reporting guidelines

This study was conducted and reported in accordance with the *STROBE* (Strengthening the Reporting of Observational Studies in Epidemiology) and *SRQR* (Standards for Reporting Qualitative Research) checklists, where applicable, to ensure clarity, transparency, and reproducibility in content analysis studies of health communication tools.

## Results

A total of 120 breast radiology and pathology reports were analyzed between January of 2023 to January of 2025. Sixty of these reports were taken from the University of Colorado, and 60 were taken from the University of Miami. At each site, 10 reports were gathered from each of the following categories: women who underwent screening mammograms, diagnostic mammograms, diagnostic breast ultrasounds, breast MRIs, percutaneous breast biopsies, and breast surgical pathology. Ninety-eight definitions were reviewed from the MedEd database using the same six readability indices. Ninety-six websites linked through the MedEd extension were also reviewed using the same six readability indices as described above.

### Readability of pathology and radiology reports

The readability of pathology and radiology reports at the University of Colorado was assessed using the six previously described readability indices, as shown in Table 1. The median Flesch-Kincaid Grade Level for all clinical reports was 11.6 (range 8–14.7), indicating that the text is generally appropriate for individuals with at least a high school education (Table 1). Other readability metrics, including the Gunning Fog Index (12.3 [8.9–17.9]), Coleman-Liau Index (15.8 [9.2–18.2]), and SMOG Index (13 [9.9–16.3]), further support the high readability level of these reports above the recommended 6 th-grade readability level. The Automated Readability Index yielded a median score of 11.2 (5.6–13.8) and the Flesch Reading Ease score was 29 (8.6–48), suggesting significant complexity and difficulty in comprehension. Subgroup analysis revealed that breast surgery pathology reports had the lowest median readability levels (range 7.0–10.9). The distribution of readability levels demonstrated that one report (1.7%) was at or below a 6 th-grade level based on the Automated Readability Index, while most reports were within a high-school or college-level comprehension for every readability formula (range 81.7%–100%).

**Table 1:** Readability values from breast pathology and radiology reports from The University of Colorado

Readability grade of pathology and radiology reports, Aim 1						
	Flesch-Kincaid Grade Level	Gunning Fog Index	Coleman-Liau Index	SMOG Index	Automated Readability Index	Flesch Reading Ease
**Type of report**						
All clinical reports	11.6 [8–14.7]	12.3 [8.9–17.9]	15.8 [9.2–18.2]	13 [9.9–16.3]	11.2 [5.6–13.8]	29 [8.6–48]
Breast biopsy pathology	10.7 [9–11.8]	12.7 [11.3–14.3]	14.3 [12.7–15.2]	12.9 [12.7–13.2]	10.3 [9.9–10.7]	38.55 [31.3–39.7]
Breast MRI	12.7 [11.6–13.3]	14.1 [12.4–16.1	16.7 [14.3–17.1]	14.1 [13.3–15.5]	12.3 [11–13.6]	23.9 [22.2–28.5]
Breast surgery pathology	9.4 [8–10.2]	9.7 [8.9–11.5]	10.9 [9.2–12.2]	10.4 [9.9–10.7]	7 [5.6–7.9]	37.7 [34.8–48]
Breast U/S	12 [11.1–14.7]	13.7 [11–17.9]	15.9 [12.7–16.5]	13.3 [12–16.3]	11.2 [9.5–13.8]	27.3 [8.6–35.7]
Diagnostic mammogram	11.8 [9.9–13]	11.3 [9.4–13.8]	16.8 [13.6–17.5]	13.4 [11.8–14.1]	12.6 [9.4–13]	28.3 [20.7–41.4]
Screening mammogram	11.6 [10.2–12.7]	11.6 [9.3–13.1]	17.1 [16.3–18.2]	12.5 [11.3–13.1]	12 [10.5–13]	25.2 [16.2–36.2]
**Readability level**						
≤ 6 th grade	0	0	0	0	1 (1.7)	0
Middle school	2 (3.3)	1 (1.7)	0	0	10 (16.7)	0
High school	38 (63.3)	26 (43.3)	9 (15)	16 (26.7)	27 (45)	0
≥ College	20 (33.3)	33 (55)	51 (85)	44 (73.3)	22 (36.7)	60 (100)

Presented values are median [range, min–max] or *n* (% frequency)

**Fig. 1:**
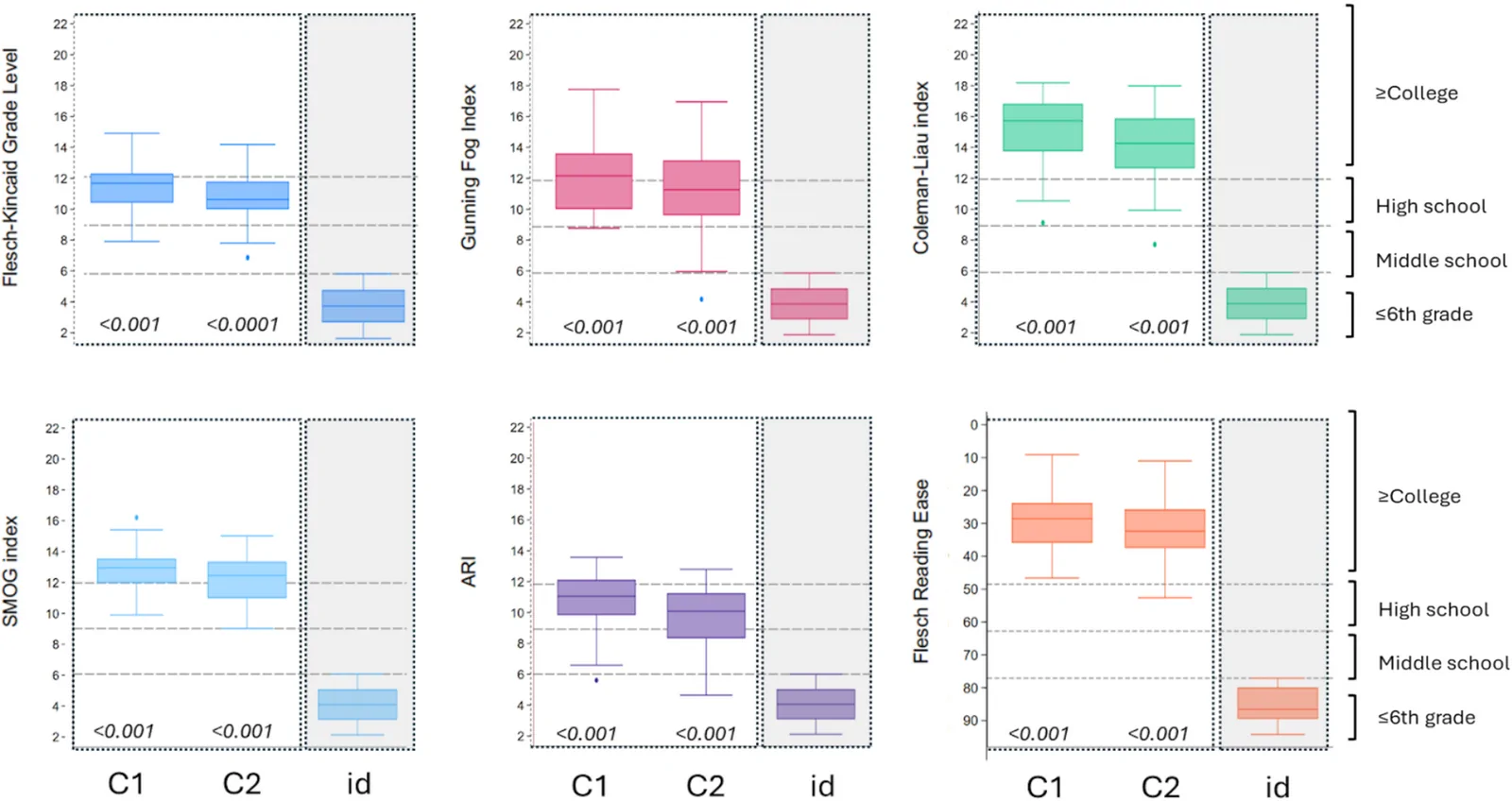
Readability of clinical reports per academic center. Scores calculated using readable.io are grouped by center and readability level. Labels: C1 = University of Colorado, C2 = University of Miami, id = ideal distribution of scores. Plotted p values show the difference of the readability between clinical reports in each center vs. the ideal distribution of readability scores (≤6th grade)

Similarly, readability scores were taken from pathology and radiology reports at the University of Miami, as shown in Table 2. Here, the grade level for all clinical reports using the Flesch-Kincaid Grade Level (10.6 [10–11.7]), Gunning Fog Index (11.4 [9.8–13.3]), Coleman-Liau Index (14.3 [12.7–15.9]), SMOG Index (12.5 [11–13.4]), Automated Readability Index (10.2 [8.4–11.4]), and Flesch Reading Ease (33 [26.1–20.3]) was calculated and determined to be at or above a 10 th-grade reading level (Table 2). Similar to the University of Colorado, on subgroup analysis, breast surgery pathology reports had the lowest median readability levels (range 7.4–11.6). The distribution of readability levels demonstrated that two reports (3.3%) were at the target readability level of less than or equal to a 6 th-grade level (Table 2). Figure 1 illustrates the significant differences in readability levels of medical reports at both the University of Colorado and Miami (C1 and C2, respectively) compared to the ideal 6 th-grade distribution (id) across all six readability indices (*p* ≤ 0.001).

**Table 2:** Readability of breast pathology and radiology reports from The University of Miami

Readability grade of clinical reports [University of Miami]						
	Flesch-Kincaid Grade Level	Gunning Fog Index	Coleman-Liau index	SMOG Index	Automated Readability Index	Flesch Reading Ease
**Type of report**						
All clinical reports	10.6 [10–11.7]	11.4 [9.8–13.3]	14.3 [12.7–15.9]	12.5 [11–13.4]	10.2 [8.4–11.4]	33 [26.1–20.3]
Breast biopsy pathology	10.3 [9.9–10.6]	11 [9.7–11.5]	14 [13.4–14.3]	12.1 [11.8–12.5]	9.6 [9–9.8]	36.5 [34.9–39.3]
Breast MRI	12.1 [11.3–12.8]	13.9 [12.9–15]	15.7 [14.8–16.3]	13.3 [12.8–13.4]	11.2 [10.4–11.4]	23.4 [21.8–31.6]
Breast surgery pathology	9.9 [9.7–10.1]	9.4 [9.1–9.9]	11.6 [11.3–11.8]	10.9 [10.7–11.1]	7.4 [7.1–7.6]	35.9 [35.1–37.1]
Breast U/S	10.9 [10.6–11.7]	12.7 [11.2–13.5]	14.7 [14.2–16]	13.3 [132–13.8]	11.3 [10.3–11.8]	33.7 [29.5–38.7]
Diagnostic mammogram	11.7 [10.9–12.4]	12.4 [10.4–13.9]	15.9 [14.8–16.1]	13.4 [12.3–14.1]	11.4 [10.5–11.8]	27.4 [25.4–32.3]
Screening mammogram	10.7 [9.4–11.7]	11.1 [9.8–13.3]	14.5 [12.6–15.8]	12.2 [10.5–13.4]	10.1 [8.2–11.4]	34.2 [30–40]
**Readability level**	Flesch-Kincaid Grade Level	Gunning Fog Index	Coleman-Liau Index	SMOG Index	Automated Readability Index	Flesch Reading Ease
≤ 6 th grade	0	1 (1.7)	0	0	2 (3.3)	0
Middle school	5 (8.3)	6 (10)	2 (3.3)	0	16 (26.7)	0
High school	42 (70)	28 (46.7)	911(18.3)	24 (40)	36 (60)	1 (1.7)
≥ College	13 (21.7)	25 (41.7)	47 (78.3)	36 (60)	6 (10)	59 (98.3)

Presented values are median [range, min–max] or *n* (% frequency)

### Readability of MedEd terminology definitions

The readability assessment of MedEd definitions (*n* = 98) indicated a median Flesch-Kincaid Grade Level of 8.7 (1.5–24.9), lower than that of clinical reports at the University of Colorado and the University of Miami (11.6 and 10.6, respectively) but still above recommended levels for patient materials (Table 3). Other readability metrics indicated moderate complexity, including a Gunning Fog Index of 10.9 (3.2–27.7), Coleman-Liau Index of 10 (4.8–18.5), and SMOG Index of 11.2 (3.1–22.1). The Automated Readability Index was 8.45 (2–30.4), suggesting increased readability compared to breast surgery pathology reports at both institutions (7.0 and 7.4, respectively). The Flesch Reading Ease score was 61.25 (0.8–98.3), indicating an 8 th–9 th grade reading level compared to the clinical reports, which were interpreted as at a college reading level (range 23.4–38.55). Compared to an ideal distribution (score at or below the 6 th-grade level), most MedEd definitions scored significantly higher readability levels (> 6 th grade, *p* = 0.0001). Depending on the index used, the percentage of definitions meeting the ideal standard ranged from 2% to 19.4% (Table 3). However, regardless of some terms falling within the ideal distribution, the median readability scores for MedEd terms across all indices were consistently above the proposed standard (*p* < 0.001 for all evaluated indices, Table 3). There was an increased distribution of readability levels in the ≤ 6 th-grade range for MedEd definitions (2.0%–19.4%) compared to the clinical reports (0.0%–3.3%). However, the majority of MedEd definitions remained above the proposed standard readability levels across all readability indexes.

**Table 3:** Readability of MedEd-directed terminology definitions

Readability grade of MedEd definitions						
	Flesch-Kincaid Grade Level	Gunning Fog Index	Coleman-Liau Index	SMOG Index	Automated Readability Index	Flesch Reading Ease
**MedEd words (*n* = 98)**						
Definitions	8.7 [1.5–24.9]	10.9 [3.2–27.7]	10 [4.8–18.5]	11.2 [3.1–22.1]	8.45 [2–30.4]	61.25 [0.8–98.3]
**Readability level**						
≤ 6 th grade	13 (13.3)	6 (6.1)	5 (5.1)	2 (2)	19 (19.4)	10 (10.2)
Middle school	40 (40.8)	16 (16.3)	30 (30.6)	55 (56.1)	35 (35.7)	26 (26.5)
High school	28 (28.6)	38 (38.8)	37 (37.8)	41 (41.8)	24 (24.5)	33 (33.7)
≥ College	17 (17.4)	38 (38.8)	26 (26.5)	0	20 (20.4)	29 (29.6)

Presented values are median [range, min–max] or *n* (% frequency)

### Readability of MedEd-directed online patient education materials

The readability assessment of OPEMs (*n* = 96) demonstrated a median Flesch-Kincaid Grade Level of 8.9 (5.2–14), comparable to MedEd definitions but still exceeding ideal patient readability thresholds (Table 4). The Gunning Fog Index was 11 (6.6–17), the Coleman-Liau Index was 10.5 (7.6–15.9), and the SMOG Index was 11.5 (8.5–15.6). The Automated Readability Index was 8.4 (5–14.8), and the Flesch Reading Ease score was 57.8 (29.9–75.4). Compared to an ideal distribution (scores at or below the 6 th-grade level), the webpages directed by MedEd demonstrated significantly higher readability scores (> 6 th grade, *p* = 0.0001). Only two web pages reported readability scores within the ideal range according to the Flesch-Kincaid Grade Level and ARI indexes (Table 4). Readability level distribution showed that only 2.1% of OPEMs were at or below a 6 th-grade level according to these indexes. Overall, these findings highlight that while MedEd definitions and OPEMs are more accessible than the clinical reports evaluated, a majority still exceed the recommended 6 th-grade reading level for patient education materials.

**Table 4:** Readability of MedEd-directed online patient education material websites

Readability grade of MedEd OPEMs						
	Flesch-Kincaid Grade Level	Gunning Fog Index	Coleman-Liau Index	SMOG Index	Automated Readability Index	Flesch Reading Ease
**MedEd words (*n* = 96)**						
OPEMs	8.9 [5.2–14]	11 [6.6–17]	10.5 [7.6–15.9]	11.5 [8.5–15.6]	8.4 [5–14.8]	57.8 [29.9–75.4]
**Readability level**						
≤ 6 th grade	2 (2.1)	0	0	0	2 (2.1)	0
Middle school	53 (55.2)	12 (12.5)	9 (9.4)	64 (66.7)	71 (73.9)	19 (19.8)
High school	39 (40.6)	67 (69.8)	77 (80.2)	32 (33.3)	21 (21.9)	68 (70.8)
≥ College	2 (2.08)	17 (17.8)	10 (10.4)	0	2 (2.1)	9 (9.4)

Presented values are median [range, min–max] or *n* (% frequency)

## Discussion

To our knowledge, this is the first study to evaluate the readability of existing breast radiology and pathology reports released at multiple institutions. This study found that readability levels of breast cancer radiology and pathology reports, as well as MedEd medical definitions and associated websites, were generally above the American Medical Association (AMA)’s recommended 6 th-grade reading level [9]. Reports at the University of Colorado and the University of Miami were written at a high school or college reading level, with a small amount at or below a middle school level. To our surprise, the MedEd tool that provides medical definitions of terms used in the reports did not meet the 6 th-grade reading level expectation. Instead, most definitions provided by the MedEd tool scored significantly higher than the recommended readability. Similarly, the MedEd tool directed patients to trustworthy websites with readability well above the 6 th-grade level. Overall, these findings highlight a significant gap between the recommended reading levels and the actual readability of individual radiology and pathology reports, MedEd definitions, and OPEMs, perpetuating barriers to patient understanding.

Many patients turn to online materials seeking information and clarification about breast cancer, but these resources often fail to meet the recommended AMA readability standards [6, 10]. Online materials may be found on general websites, such as Google or Wikipedia, that not only struggle to meet readability standards but may also provide inaccurate or misleading medical information [10]. In contrast, the MedEd tool purposefully redirects patients toward accurate, reliable websites with evidence-based sources. The gap between actual readability and recommended standards can cause confusion and anxiety for patients accessing their reports independently through a patient portal, especially before discussing these results with their healthcare providers [11]. In a 2024 study by O’Brien et al., with the immediate release of patient records, patients and clinicians alike were open to supplemental resources being included with their individual reports to help with overall comprehension [22]. Such misunderstanding may affect a patient’s ability to make informed decisions and feel in control of their healthcare. Our study aimed to highlight the readability of breast cancer radiology and pathology reports, emphasizing the need to make these reports more user-friendly and easier to understand so patients can engage in shared decision-making and better manage their health.

In 2024, over 42 million women underwent mammography, and over 1 million underwent breast biopsies [15, 16]. Given the high volume of patients receiving radiology or pathology reports from these services, our study underscores the critical need to bridge the gap between patient-accessible medical reports and an appropriate understanding of the medical terminology within these reports. This may lead to improved comprehension and informed decision-making.

### Strengths

One of the key strengths of our study is that it is the only paper to our knowledge that directly examines individualized breast pathology and radiology reports, allowing us to assess real-time readability challenges as patients receive their results through patient portals. Unlike prior studies that rely on hypothetical scenarios or general readability assessments, our analysis captures the complexity of the language patients encounter firsthand [12, 14]. Additionally, our study is a multi-institutional analysis, including 120 unique radiology and pathology reports from institutions in both Colorado and Florida. This geographic diversity strengthens the generalizability of our findings, suggesting that poor readability is a widespread issue rather than a localized problem. We believe our novel MedEd plug-in is a key strength, offering a powerful tool to bridge the readability gap in patient education. This plug-in provides a controlled, reliable way to deliver trusted, healthcare-vetted information, ensuring patients receive accurate and accessible knowledge—unlike the uncertainty of searching for credible sources on their own.

### Limitations

A major limitation of this study is the lack of direct patient feedback, which is crucial for accurately assessing comprehension. However, a study by Foy et al. in 2024 demonstrated acceptability, feasibility, and comprehension of MedEd with the use of direct patient feedback [21]. The use of readability scoring tools alone may fail to capture understanding, as they tend to rely on elements such as sentence length, syntax, and terminology. They do not reflect how an individual may understand the information in the broader context in which it is presented. To address this limitation in future research, we plan to incorporate artificial intelligence (AI) to ensure reports meet the 6 th-grade readability level and gather direct patient feedback to compare and contrast original reports to the revised versions. These tools also do not account for diverse health literacy among patients. The readability of pathology and radiology reports may be better assessed with the involvement of direct patient feedback, such as surveys or interviews. Cultural and language differences, which were not fully accounted for in this study, also play a significant role in comprehension. Non-native English speakers may have more difficulty understanding reports in English, resulting in comprehension barriers [17, 18]. In future studies, we hope to incorporate Spanish AI versions of the reports through the MedEd tool to better address this limitation and improve accessibility among diverse populations. Finally, in an attempt to improve readability, medical information may be oversimplified. This may lead to the omission of critical information that can be crucial for patient decision-making. Despite these limitations, this is a novel study utilizing six validated readability indices to objectively understand the education level required to understand real, unique, breast radiology and pathology reports.

The significant gap in readability levels between the recommended 6 th-grade level and the actual readability poses a major barrier for patients to understand their own health. When health reports are too complex, patients may struggle to comprehend critical details, resulting in confusion, fear, and unnecessary anxiety [19]. A lack of understanding of health information creates a struggle to make informed decisions and may lead to disengagement in a patient’s own care [20]. As a result, patients may be less likely to adhere to treatment plans, negatively impacting health outcomes [20]. There is a clear need for innovation, such as using AI to simplify radiology and pathology reports to at or below a 6 th-grade reading level, while maintaining the integrity of the medical information in such reports. Future studies will address the clear need for innovation by exploring the use of AI to simplify radiology and pathology reports to at or below a 6 th-grade reading level while maintaining the integrity of the medical information within these reports. By addressing the readability disparity, we can increase patient engagement, decrease confusion, and improve overall health outcomes, ensuring patients are equipped with the tools needed to navigate their health.

## Conclusion

In conclusion, our study demonstrates that a majority of breast radiology and pathology reports are above the recommended 6 th-grade reading level by six validated readability indices. Additionally, while OPEMs and MedEd-provided texts showed improved accessibility compared to the original medical reports, a majority exceeded the recommended 6 th-grade reading level for patient education materials. This highlights a persistent need for patient-centered materials at appropriate reading levels to support patients with the independent interpretation of their medical reports. Future work should focus on the development of tools that can be used by patients while they review their result reports independently.

## Data Availability

No datasets were generated or analysed during the current study.

## References

[1] Cho YI, Lee SY, Arozullah AM, Crittenden KS. Effects of health literacy on health status and health service utilization amongst the elderly. Soc Sci Med. 2008;66(8):1809–16. 10.1016/j.socscimed.2008.01.003 18295949

[2] Williams MV, Davis T, Parker RM, Weiss BD. The role of health literacy in patient physician communication. Fam Med. 2002;34(5):383–9. 12038721

[3] Mityul MI, Gilcrease-Garcia B, Searleman A, Demertzis JL, Gunn AJ. Interpretive differences between patients and radiologists regarding the diagnostic confidence associated with commonly used phrases in the radiology report. AJR Am J Roentgenol. 2018J;210(1):123–6. 10.2214/AJR.17.18448. Epub 2017 Oct 12 PMID: 29023151. 29023151

[4] Weiss BD. Health literacy and patient safety: help patients understand. 2nd ed. Chicago, IL: American Medical Association Foundation; 2007.

[5] Gu JZ, Baird GL, Escamilla Guevara A, Sohn YJ, Lydston M, Doyle C, Tevis SEA, Miles RC. A systematic review and meta-analysis of English language online patient education materials in breast cancer: is readability the only story? Patient Educ Couns. 2024A 10;S0960–9776(24):00053–5. 10.1016/j.pec.2024.107006. Epub 2024 Apr 3. PMC11019273 38603836

[6] Lamb, Leslie R., et al. “Are English-language online patient education materials related to breast cancer risk assessment understandable, readable, and actionable?” The Breast, vol. 61, Feb. 2022, pp. 29–34, 10.1016/j.breast.2021.11.012. Accessed 6 Feb. 2022. PMC8665407 34894464

[7] Pew Research Center. Health online 2013. Washington, DC: Pew Research Center; 2013 [updated 2013 Jan 15]. Available from: https://www.pewresearch.org/internet/wp-content/uploads/sites/9/media/Files/Reports/PIP_HealthOnline.pdf.

[8] Miller, David P, et al. Primary care providers’ views of patient portals: interview study of perceived benefits and consequences. Journal of Medical Internet Research, U.S. National Library of Medicine, 15 Jan. 2016, www.ncbi.nlm.nih.gov/pmc/articles/PMC4733220/. 10.2196/jmir.4953 PMC4733220 26772771

[9] Weiss BD. Health literacy: a manual for clinicians. American Medical Association Foundation and American Medical Association; 2003.

[10] Valencia EA, Cortina CS, Cobb AN, Chaudhary LN, Kelly T, Kong AL. Evaluating the accuracy, quality, and readability of online breast cancer information. Ann Surg Open. 2024J 15;5(3). 10.1097/AS9.0000000000000465. PMC11415127 39310356

[11] Giardina TD, Baldwin J, Nystrom DT, Sittig DF, Singh H. Patient perceptions of receiving test results via online portals: a mixed-methods study. J Am Med Inform Assoc. 2018A;25(4):440–6. 10.1093/jamia/ocx140. PMC5885801 29240899

[12] Verosky A, Leonard LD, Quinn C, Vemuru S, Warncke E, Himelhoch B, Huynh V, Wolverton D, Jaiswal K, Ahrendt G, Sams S, Lin CT, Cumbler E, Schulick R, Tevis SE. Patient comprehension of breast pathology report terminology: the need for patient-centered resources. Surgery. 2022S;172(3):831–7. 10.1016/j.surg.2022.05.007. 35715235

[13] O'Brien E, Ludwigson A, Vemuru S, Higgins M, Hampanda K, Adams M, Wolverton D, Sams S, Taft N, Miles R, Lin CT, Cumbler E, Tevis S. Interpretation of immediately released health information: informing patient medical education in breast oncology. Am J Surg. 2025 Jan;239:115853. 10.1016/j.amjsurg.2024.115853. Epub 2024 Jul 18. PMID: 39095250; PMCID: PMC11649473. PMC11649473 39095250

[14] Martin-Carreras T, Cook TS, Kahn CE Jr. Readability of radiology reports: implications for patient-centered care. Clin Imaging. 2019 Mar-Apr;54:116–120. 10.1016/j.clinimag.2018.12.006. 30639521

[15] U.S. Food & Drug Administration. MQSA National Statistics [Internet]. Silver Spring, MD: U.S. Food & Drug Administration; 2025 [updated 2025 Jan 2; cited 2025 Feb 22]. Available from: https://www.fda.gov/radiation-emitting-products/mammography-information-patients/mqsa-national-statistics.

[16] Agency for Healthcare Research and Quality. Core-needle biopsy for breast abnormalities [Internet]. Rockville, MD: Effective Health Care Program, Agency for Healthcare Research and Quality; 2016 [updated 2014; cited 2025 Feb 25]. Available from: https://effectivehealthcare.ahrq.gov/products/breast-biopsy-update/clinician.

[17] Berland GK, Elliott MN, Morales LS, Algazy JI, Kravitz RL, Broder MS, Kanouse DE, Muñoz JA, Puyol JA, Lara M, Watkins KE, Yang H, McGlynn EA. Health information on the Internet: accessibility, quality, and readability in English and Spanish. JAMA. 2001M 23;285(20):2612–21. 10.1001/jama.285.20.2612.PMID:11368735;PMCID:PMC4182102. PMC4182102 11368735

[18] Chu JN, Sarkar U, Rivadeneira NA, Hiatt RA, Khoong EC. Impact of language preference and health literacy on health information-seeking experiences among a low-income, multilingual cohort. Patient Educ Couns. 2022M;105(5):1268–75. 10.1016/j.pec.2021.08.028.PMID:34474924;PMCID:PMC9205365. PMC9205365 34474924

[19] Evidence NIHR. Health information: are you getting your message across? NIHR Evidence. 2022J. 10.3310/nihrevidence_51109.

[20] Martin LR, Williams SL, Haskard KB, DiMatteo MR. The challenge of patient adherence. Ther Clin Risk Manag. 2005 Sep;1(3):189–199. PMID: 18360559; PMCID: PMC1661624. PMC1661624 18360559

[21] Foy B, Ludwigson A, Mott NM, Adams M, Higgins MG, Vemuru S, Wolverton D, Yi J, Sams S, Lin CT, Miles R, Taft N, Baurle E, Cumbler E, Tevis S. Helping patients navigate the immediate release of medical records: MedEd, a novel patient engagement technology. Am J Surg. 2025J;239. 10.1016/j.amjsurg.2024.09.016. 39322528

[22] O’Brien E, Vemuru S, Leonard L, Himelhoch B, Adams M, Taft N, Jaiswal K, Sams S, Cumbler E, Wolverton D, Ahrendt G, Yi J, Lin CT, Miles R, Hampanda K, Tevis S. Information transparency with immediate release: oncology clinician and patient perceptions. Am J Surg. 2024J;227:165–74. 10.1016/j.amjsurg.2023.10.015.PMID:37863801;PMCID:PMC11112620. PMC11112620 37863801

[23] Kincaid JP, Fishburne RP Jr, Rogers RL, Chissom BS. Derivation of new readability formulas for navy enlisted personnel. Res Branch Rep. 1975 Feb;8–75:1–39. Available from: https://apps.dtic.mil/sti/pdfs/ADA006655.pdf.

[24] Flesch R. A new readability yardstick. J Appl Psychol. 1948J;32(3):221–33. 10.1037/h0057532. 18867058

[25] Gunning R. The technique of clear writing. New York: McGraw-Hill; 1952.

[26] Coleman M, Liau TL. A computer readability formula designed for machine scoring. J Appl Psychol. 1975A;60(2):283–4. 10.1037/h0076540.

[27] McLaughlin GH. SMOG grading: a new readability formula. J Read. 1969M;12(8):639–46.

[28] Senter RJ, Smith EA. Automated Readability Index. Wright-Patterson Air Force Base, OH: Aerospace Medical Research Laboratories; 1967 Feb. AMRL-TR-66–220. Available from: https://apps.dtic.mil/sti/pdfs/AD0667273.pdf. 5302480

